# Supplementary material for: Gold@Silica Nanoparticles Functionalized with Oligonucleotides: A Prominent Tool for the Detection of the Methylated Reprimo Gene in Gastric Cancer by Dynamic Light Scattering
Source: Nanomaterials (Basel). 2019 Sep 18;9(9):1333. doi: 10.3390/nano9091333 (PMC6781027; doi:10.3390/nano9091333)
Supplement: Supplementary file 1 [file nanomaterials-09-01333-s001.pdf]

nanomaterials

Article

Gold@Silica Nanoparticles Functionalized with Oligonucleotides: A Prominent Tool for the Detection of the Methylated Reprimo Gene in Gastric Cancer by Dynamic Light Scattering

María José Marchant [1], Leda Guzmán [1], Alejandro H. Corvalán [2,3] and Marcelo J. Kogan [3,4,*]

[1] Laboratorio de Química Biológica, Instituto de Química, Pontificia Universidad Católica de Valparaíso, 2373223 Valparaíso, Chile; marchant.mariajose@gmail.com (M.J.M.); leda.guzman@pucv.cl (L.G.)
[2] Departamento de Hematología y Oncología, Facultad de Medicina, Pontificia Universidad Católica de Chile, 8330032 Santiago, Chile; acorvalan@uc.cl
[3] Advanced Center for Chronic Diseases (ACCDiS), Pontificia Universidad Católica de Chile, 8330034 Santiago, Chile
[4] Departamento de Química Farmacológica y Toxicológica, Facultad de Ciencias Químicas y Farmacéuticas, Universidad de Chile, 8380494 Independencia, Santiago, Chile
* Correspondence: mkogan@ciq.uchile.cl; Tel.: +56-9-8903-4877

Received: 20 July 2019; Accepted: 12 September 2019; Published: date

Supplementary file

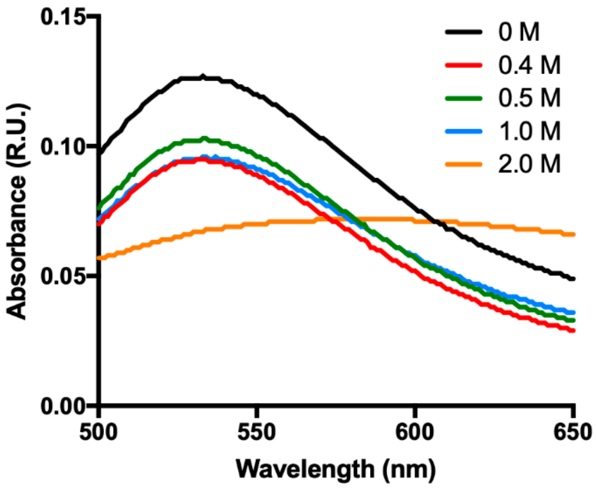

Figure S1. Stability assay of Au@SiO 2 -COOH-Oligo with NaCl. Absorption spectra for Au@SiO2-COOH-Oligo in presence of different concentrations of NaCl. A redshift is observed at 2.0 M NaCl due to partial aggregation Au@SiO2-COOH-Oligo.

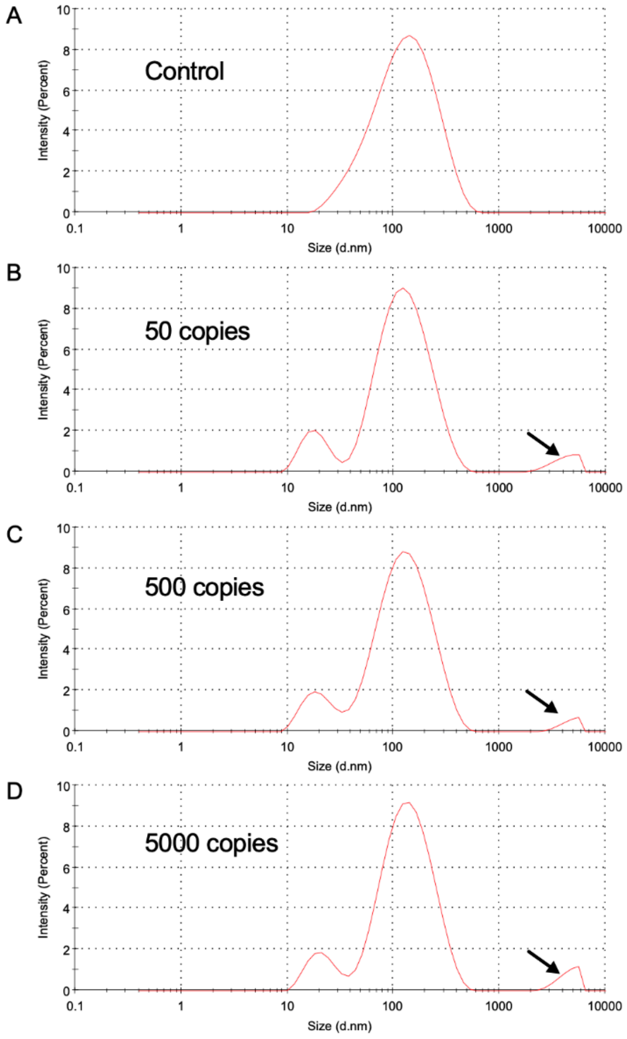

Figure S2. DLS measurements of Au@SiO2-COOH-Oligo nanoparticles after the hybridization assay with the synthetic fragment of the methylated RPRM DNA. Nanoparticles were incubated with methylated RPRM DNA in PBS1X for 30 minutes at 37ºC with vigorous stirring. One peak (indicated with black arrow) close to 4000 nm is observed in B, C and D, and is attributable to the formation of hybrids between Au@SiO2-COOH-Oligo nanoparticles and methylated RPRM DNA, when compared to control assay without DNA (A). The profiles are representative of the hybridization assays realized. The y axis indicates the intensity based on the weights of dispersed materials.

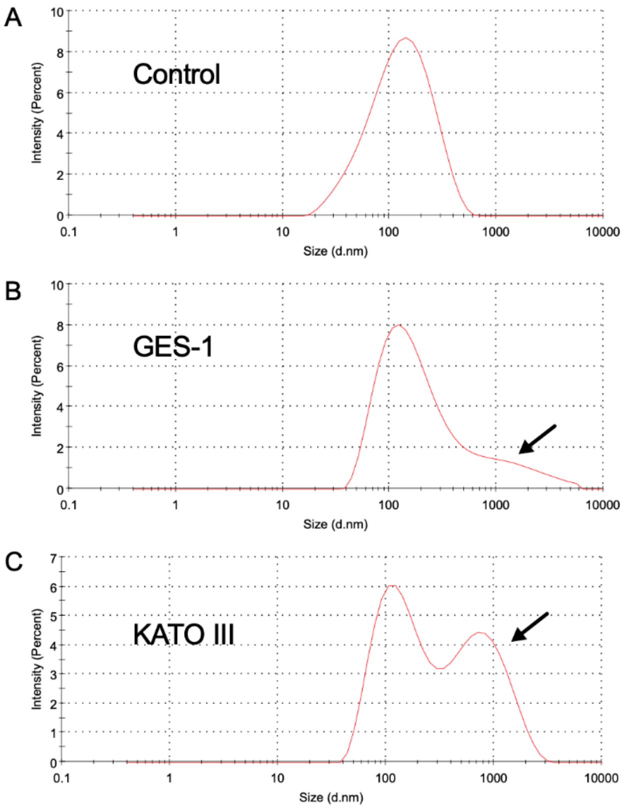

Figure S3. DLS measurements of Au@SiO_2-COOH-Oligo nanoparticles after the hybridization assay with genomic DNA cell lines. Nanoparticles were incubated with methylated RPRM DNA in PBS1X for 30 minutes at 37ºC with vigorous stirring. A. The control reaction without DNA. B. The reaction with genomic DNA from the GES-1 cell line. C. The reaction with genomic DNA from the KATO III cell line. A shoulder peak (indicated with black arrow) close to 1000 nm is observed in B and is attributable to the partial aggregation of Au@SiO2-COOH-Oligo when compared to control assay without DNA (A). In the case of C, one peak attributable to the formation of hybrids between Au@SiO2-COOH-Oligo and methylated RPRM DNA is observed when compared to the control assay without DNA (A). The profiles are representative of the hybridization assays realized. The y axis indicates the intensity based on the weights of dispersed materials.

© 2019 by the authors. Submitted for possible open access publication under the terms and conditions of the Creative Commons Attribution (CC BY) license (http://creativecommons.org/licenses/by/4.0/).
